# Development of Calibration Models to Predict Mean Fibre Diameter in Llama (*Lama glama*) Fleeces with Near Infrared Spectroscopy

**DOI:** 10.3390/ani11071998

**Published:** 2021-07-04

**Authors:** José Ignacio Amorena, Dolores María Eugenia Álvarez, Elvira Fernández-Ahumada

**Affiliations:** 1 Instituto Nacional de Tecnología Agropecuaria (INTA) Estación Experimental Agropecuaria (EEA) Catamarca, RP N° 33, km 4.5, Catamarca 4705, Argentina; 2 Centro de Investigación y Tecnología Química (CITeQ) (CONICET-UTN), Maestro Marcelo López esq. Cruz Roja Argentina, Ciudad Universitaria, Córdoba 5016, Argentina; dalvarez@frc.utn.edu.ar; 3 Departamento de Matemáticas, Universidad de Córdoba (UCO), 14071 Córdoba, Spain; g82feahe@uco.es

**Keywords:** llama, textile fibres, near infrared spectroscopy, NIRS

## Abstract

**Simple Summary:**

In the Puna region of Argentina, llama fibre production has enormous social, economic and environmental potential, but is still in its early stages of development. For this reason, classification and quality analysis systems used today are still deficient. Near infrared reflectance spectroscopy is a technological resource used in the agroindustry for quality analysis of organic compounds. In this work we studied the feasibility of this technology to evaluate the mean fibre diameter, which is one of the most important quality parameters in the textile industry. Despite some limitations, which are mainly related to fibre heterogeneity, the results obtained were encouraging as spectroscopy could be used in screening programmes as a sustainable, fast and low-cost method to improve fibre quality validation.

**Abstract:**

Llama fibre has the potential to become the most valuable textile resource in the Puna region of Argentina. In this study near infrared reflectance spectroscopy was evaluated to predict the mean fibre diameter in llama fleeces. Analyses between sets of carded and non-carded samples in combination with spectral preprocessing techniques were carried out and a total of 169 spectral signatures of llama samples in Vis and NIR ranges (400–2500 nm) were obtained. Spectral preprocessing consisted in wavelength selection (Vis–NIR, NIR and discrete ranges) and multiplicative and derivative pretreatments; spectra without pretreatments were also included, while modified partial least squares (M-PLS) regression was used to develop prediction models. Predictability was evaluated through R^2^: standard cross validation error (SECV), external validation error (SEV) and residual predictive value (RPD). A total of 54 calibration models were developed in which the best model (R^2^ = 0.67; SECV = 1.965; SEV = 2.235 and RPD = 1.91) was obtained in the Vis–NIR range applying the first derivative pretreatment. ANOVA analysis showed differences between carded and non-carded sets and the models obtained could be used in screening programs and contribute to valorisation of llama fibre and sustainable development of textile industry in the Puna territory of Catamarca. The data presented in this paper are a contribution to enhance the scarce information on this subject.

## 1. Introduction

The textile industry is one of the main polluting industries worldwide; it generates 13 million tonnes of plastic waste per year [1]. In this regard, natural fibres (animal, vegetable and mineral) play an essential role as a sustainable alternative to reduce the pollution generated by this activity. Animal fibres are characterised by their insulating capacity, thermal balance, durability, quality and commercial prestige [2]. These fibres are divided into two main categories: sheep wool and speciality fibres, which are obtained from hair shearing of goats (mohair and cashmere), camelids (camel, alpaca, guanaco, llama and vicuña) and other animals (silk, angora rabbit, etc.).

The Puna region is the largest, least inhabited and poorest place on the American Continent. It is characterised by its altitude (above 3000 m.a.m.s.l.), rugged geography and extreme climatic conditions [3]. In this hostile environment, domestic South American camelids (llama, *Lama glama* and alpaca, *Lama pacos*) and wild camelids (guanaco, *Lama guanicoe* and vicuña, *Vicugna vicugna*) have been able to adapt successfully, becoming the main resource for meat and clothing [4,5]. In the Puna territory of Argentina (Catamarca, Jujuy and Salta provinces), llama fibre is emerging as a promising production alternative in the specialty fibre industry due to its textile characteristics and sustainable production [6].

Animal fibres in general and llama in particular present a complex and heterogeneous profile. This is due to its physicochemical composition such as keratin structures [7], morphology and fleece conformation (fibres that constitute a fleece are highly entangled) [8] and the presence of extraneous particles such as plant debris, dust, sand, etc. Numerous methods are used by the textile industry for fibre refinement [9] and fibre carding is considered essential in the early stages of this process since it is used for cleaning, untangling and homogenising the fleeces [10,11].

Several quality parameters are quantified throughout textile industrial processing, among which the mean fibre diameter (MFD) is the most important [12]. MFD indicates the cross-sectional dimension of fibres constituting the fleece. This feature, usually expressed in micrometres (µm), is the main criterion for determining price, processing performance and end-use in most animal fibres [13]. The projection microscope, airflow, Laserscan and optical fibre diameter analyser (OFDA) [14] are the classical and most commonly employed methods for measuring MFD. However, some of these methods are contaminant, slow, destroy the sample and require user prior training [15].

Over the years, near infrared reflectance spectroscopy (NIRS) has become one of the most widely used methods for quality evaluation in the agricultural sector. NIRS is a fast, low-cost and sustainable technique that allows the analysis of multiple parameters simultaneously with minimal sample preparation. Its functioning is based on a light interaction with the sample to be analysed. As a result of this interaction an absorption spectrum is obtained. A NIR spectrum covers the wavelength range of 780–2500 nm and is related to overtones and combinations of fundamental vibrations of hydrogen-containing groups such as S-H, C-H, N-H and O-H [16]. Spectra obtained are correlated by means of regression methods with their corresponding chemical composition parameters and other quantitative or qualitative attributes in a procedure known as calibration or prediction model development [17]. Model performance is evaluated via statistical variables such as the coefficient of determination, bias, prediction errors and ratio between error and standard deviation (RPD) [18], which provides an outline of possible practical applications of any given model. A crucial aspect to consider in reflectance spectroscopy is light scattering. This phenomenon is strongly related to the analyte structure [19] and occurs when light interacts with particles of different size and shape and is reflected backwards in all directions [20]. In the case of heterogeneous analytes such as animal fibres, the scattering effect will be greater. When a fibre sample is analysed by spectroscopy, light scattering effects prevail, thus low spectral repeatability is obtained. This entails inconveniences in developing accurate prediction models compared to homogeneous samples, such as flours or chopped and grounded forages [21]. To remove the variability that may be caused by scattering, derivative and multiplicative spectral preprocessing are commonly applied [22].

Several reports were found on the use of NIRS technology to predict textile fibre-quality parameters. Applications have focused on yield estimations [23], ash and grease content determination [24], fibre origin classification [16,25], measurement of medullated fibres [26] and lustre identification in alpaca fibres [27]. Few studies have reported the use of NIRS to analyse MFD in wool [24,28] and alpaca fibre [13,29]. These reports assume that results, although encouraging, have not achieved the accuracy required to be used by industry, especially regarding quantitative parameters such as MFD.

The objective of this study is to evaluate the feasibility of NIR spectroscopy to develop predictive models of MFD in llama fibre samples. For this purpose, analyses between sets of carded and non-carded samples in combination with spectral preprocessing techniques were carried out. These techniques included wavelength selection and multiplicative and derivative processing. Treatment performance was evaluated by comparing prediction statistical variables from each calibration model developed for MFD on llama fleece samples.

The remarkable potential of natural fibres, a lack of resources in the Puna territory of Argentina and consumer demand for knowing the origin and manufacturing processes of their garments, gives the opportunity to add value to local textile fibre production. From this perspective, NIR spectroscopy could be a valuable contribution allowing quality improvement by providing reliable information to consumers and contributing to the positioning of llama yarns and garments in the textile market.

## 2. Materials and Methods

### 2.1. Samples: Characterisation and Treatments

A total of 169 llama samples of seven different fleece colours (white, black, grey, brown, light brown, coffee and beige) were collected from 3 sites in the Puna region of Catamarca province: Antofagasta de la Sierra, Laguna Blanca and Santa María. Each sample (2 g approximately) was obtained by cutting a portion from the mid-side of the animal fleece with ordinary scissors, then placed in a labelled plastic bag and stored until analysis [30]. Llama fibre was classified by 5 fineness ranges: baby < 19 µm, superfine 19–21.9 µm, fine 22–24.9 µm, medium 25–29 µm and coarse > 30 µm [31]. This classification method was adapted from the Peruvian alpaca classification system. Sampling was oriented to cover the widest range of MFD variability present throughout the territory.

Samples were carded with a special brush in order to disentangle, parallelise and homogenise fibres; dust, sand and plant debris (thistles, thorns, straws, etc.) were also removed [32].

### 2.2. Reference Analysis

In order to obtain reference data to be used in NIR spectroscopy calibration, snippets from each sample were cut and then analysed using the Optical Fibre Diameter Analyser 2000 (OFDA 2000) [14]. This instrument determines the fibre diameter distribution by measuring individual fibres of snippets through an image digitalisation programme [33]. The parameters analysed were: mean fibre diameter (MFD) and standard deviation of MFD (SDMFD), both values expressed in micrometres; coefficient of variation of MFD (CVMFD), which is the percentage of the relationship between SDMFD and MFD; comfort factor (CF), which is the number of fibres less than and equal to 30 µm, divided by the total number of fibres, expressed as a percentage [34,35].

### 2.3. Spectra Collection

Samples were placed in a 12 cm diameter circular sample holder and covered with a metal lid for scanning. Spectra were collected with a DS2500 NIRS spectrophotometer (FOSS Analytical Systems, Nils Foss Alé 1, Hillerød, Denmark) in the visible (400–780 nm) and near infrared (780–2500 nm) spectral ranges, in an interval of 0.5 nm (4200 datapoints). Three spectra were taken on each fibre sample (repack) [29,36]. An average spectrum of these three measurements was used for subsequent spectral processing. Spectra were collected through ISIscan Nova and Mosaic software (FOSS A/S, Nils Foss Alé 1, Hillerød, Denmark) and stored as absorbance units (A = log1/R), where R corresponds to reflected energy [37]. All samples were scanned twice; first, the samples were scanned as obtained from the animal; then, these same samples were carded and scanned again and spectra obtained were saved as the control and carded set, respectively.

### 2.4. Spectral Preprocessing and Calibration Models Development

Multivariate data analysis software WinISI ver. 4.10 (Infrasoft International LLC.1362 South Atherton St., State College, PA 16801, USA) was used for spectral preprocessing and calibration model development.

Wavelength selection: spectra of a sample are usually a series of intensity values of hundreds of wavelengths. In some cases, not all of them are equally important for modelling. Three datasets with varying spectral ranges were used:

- Vis–NIR: range from 400 to 2500 nm (4200 datapoints).
- NIR: range from 1100 to 2500 nm (2800 datapoints). Visible and a section of the NIR region of the spectra was discarded due to a large variability originated by pigmented fibres.
- Discrete: range containing a set of absorption bands of specific composites in animal fibres (2300 datapoints) [7,13,38].

Derivative pretreatments: derivative treatments used in this work were: 0-0-1-1, 1-5-3-1 and 2-5-3-1. The first digit indicates the order of the derivative, the second specifies the number of terms of the derivative equation and the last two designate the number of terms used for smoothing.

Multiplicative pretreatments: weighted multiplicative scatter correction (WMSC) and standard normal variate and detrend (SNVD) were applied. In both, least squares regression was used to compare the value of each individual spectrum, either against the mean value of total spectra (WMSC) or against the mean value of itself (SNVD) [38]. In addition, a set of spectra without multiplicative treatments (NONE) was included [22].

Calibration models: mean fibre diameter reference data were used for the calibration model development. Regression method, known as modified-partial least squares (M-PLS), was used [28]. This method combines the generalised principal component analysis and multilinear regression [39]. It creates an orthogonal latent variable from the spectra and then identifies the relationship from the latent variables to the reference variables [40].

From all 169 samples, 127 samples were used as a calibration set for prediction model development. The remaining 42 samples were used for external validation of the prediction models [41]. Samples were selected according to Mahalanobis distance H [42]. Additionally, cross-validation was used to test the accuracy of the calibration in each step. Cross-validation was performed by dividing each population from the calibration set into five groups. Then, values of each group were predicted using the calibration developed from the remaining groups [43].

The predictive performance of each model was evaluated on the basis of the following statistical variables: coefficient of determination (R^2^), standard error of cross validation (SECV), standard error of external validation (SEV) and relative predictive determinant (RPD). RPD indicates model accuracy and represents the ratio between standard deviation of the reference analysis and SECV [27,44]. In order to select the best calibration models, the RPD value greater than three has been considered adequate for analytical purposes. On the other hand, values lower than three, indicates that models can be used for classification (ranking) purposes [13,29]. Additionally, the maximum R^2^ value and minimum validation errors (SECV and SEV) were considered. All these variables were compared by means of ANOVA analysis to determine whether there was any significant difference between control and carded treatments.

## 3. Results

### 3.1. Reference Analysis

Table 1 shows statistical parameters from the reference data of 169 llama samples. Wide variability between minimum and maximum values in all variables were observed. This feature is characteristic in domestic camelids such as llama [45] and alpaca [46]. According to data obtained on the MFD reference analysis, 51% of samples corresponded to fine (*n* = 54), superfine (*n* = 26) and baby (*n* = 6), while 38% (*n* = 64) corresponded to the medium type and 11% (*n* = 19) to coarse.

**Figure 1:**
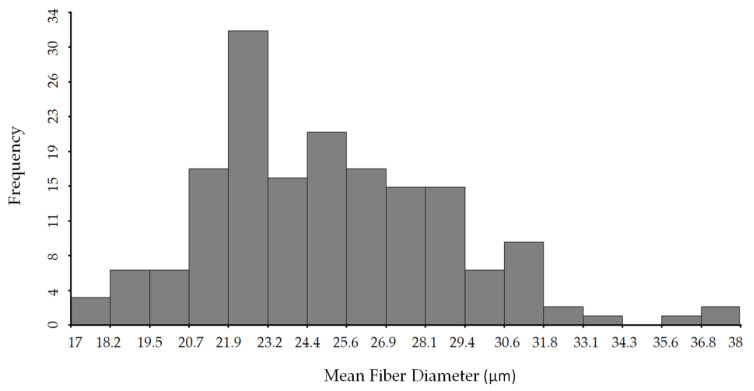
Frequency distribution of MFD values from reference analysis.

Sample distribution is a key component in obtaining accurate prediction models since it must represent all possible variations within a given parameter. In other words, the frequency distribution for any parameter should have a flattened or rectangular shape. Figure 1 shows the frequency distribution of the MDF parameter. In this case the histogram has a right skewed distribution-like shape.

### 3.2. Spectra Collection

**Figure 2:**
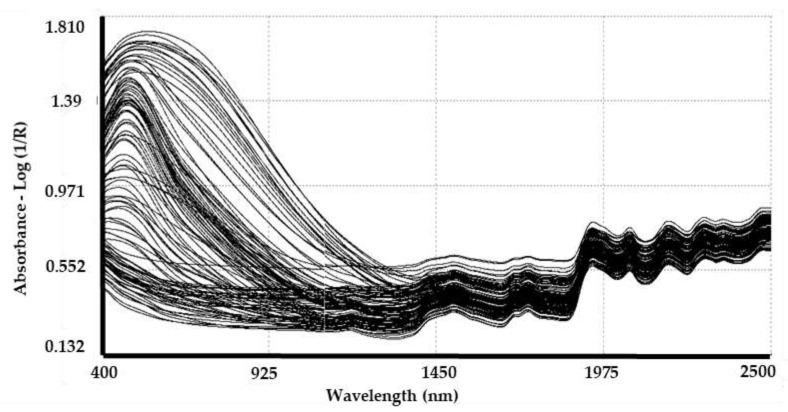
Visible and near infrared reflectance from raw spectra of all samples. Axis: absorbance (log 1/R) vs. VIS–NIR wavelength (400–2500 nm).

Figure 2 shows the 169 raw spectra corresponding to the control set samples. Absorbance spectra of the whole sample set shows a remarkable variability in the VIS segment (400–850 nm) caused by fleece colours [13]. From 850 to 1400 nm, the variability observed is characteristic of pigmented fibres and is related to absorptions of melanin pigments [47]. From 1400 to 2500 nm, the variability decreased and absorption bands from overtones and combinations of C–H, N–H, O–H and S–H bonds that originated in the mid infrared region can be observed.

**Figure 3:**
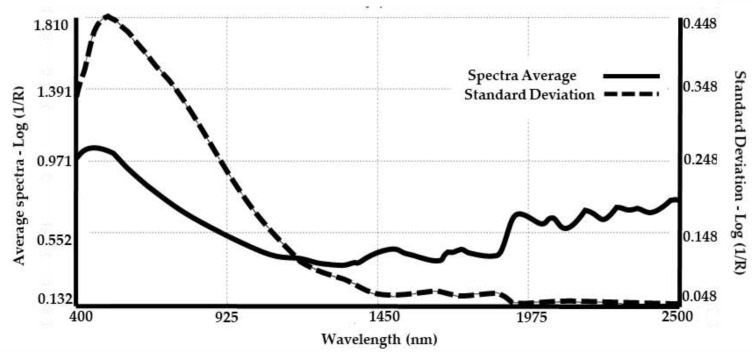
Average spectra (**right** *y*-axis) and standard deviation (**left** *y*-axis) of llama fibre samples.

Average and standard deviation of all spectra (400–2500 nm) are shown in Figure 3. Standard deviation shows prominent values in the 400–1400 nm region explained by the colour diversity of the samples. From 1400 to 2500 nm, smaller variations can be noted related to the main absorption bands in the average spectra [13]. The average spectra show absorption bands at 1450 nm assigned to the first overtone of the O–H stretching vibration of water. Bands around 1900 nm were assigned to combinations of the O–H stretch and H–O–H bending vibrations of the hydroxyl group from water. Double peaks around 1700 nm were associated with the first C–H stretch overtones of lipids and protein side chains. The segment between 2000 and 2500 nm provides information on amino acid composition and molecular conformation of animal keratin fibres. For example, absorption bands around 2055 nm, 2160 nm and 2290 nm are related to combination bands of N-H groups present in the α-helix and β-sheet structure of keratin [48].

### 3.3. Spectral Processing and Calibration Models Analysis

A total of 54 calibration models were developed applying combinations between sample treatment and spectral processing. Table 2 shows the best predictive models selected by means of highest R^2^ and RPD, and minimum validation errors, for each spectral range and sample treatment. Models 1, 10 and 19 from the control set were obtained without applying multiplicative or derivative pretreatments (NONE 0011). On the other hand, models 29, 37 and 46 from the carded set were obtained similarly to those from the control, except model 29, which was obtained using the first derivative pretreatment. The ANOVA analysis of statistical variables (R^2^, RPD, SECV and SEV) showed significant differences between carded and control samples. The information about all calibration models obtained is available in Table S1 of the Supplementary Materials.

## 4. Discussion

### 4.1. Reference Analysis

The development of the llama textile industry in Argentina is in its early stages of development compared to the sheep wool industry or alpaca fibre production in countries such as Peru or Bolivia. This results in a low level of technological input into the production system.

In this section, reference data obtained was compared with analyses of llama, alpaca and sheep wool. The MFD mean value shown in Table 1 (25.16 µm) was similar to those reported by Frank and Hick [49] but higher than 23.9 µm obtained by Mueller et al. [6] in Catamarca. In Southern and Central Argentina, values of MDF obtained were 25 µm [6], 26.2 µm [50] and 28.7 µm [11]. Moreover, the minimum (17.82 µm) and maximum (37.71 µm) values were slightly different than values reported by Laime Huarcaya et al. [45] for Peruvian llamas (16.18 µm and 41.42µm) and 16.4 µm and 34.2 µm reported by Canaza-Cayo et al. [13] in alpaca fibres. Several reports have established that average fineness in Argentinian llama fibres is about 23 µm [6,31,47,51]. Similar values were reported in Bolivia and Perú [45]. It must be noted that all values mentioned correspond to analyses made on raw fibres.

In this work, CVMDF represents uniformity in the values of MFD distribution. The CVMDF obtained (33.46%) exceeds the estimated value for the textile yield (24%) [46]. Mueller et al. reported CVMDF values of 29.2% in Catamarca and 26.5% in South and Central Argentina. Wurzinger et al. [52] obtained CVMDF values of 33.1% in Bolivia, and Laime Huarcaya reported CVMDF of 22.14% for Peruvian llamas [45]. Furthermore, the analysis made on alpaca fibres, Canaza-Cayo and Gishen and Cozzolino reported a CVMDF of 23.55% and 24.95% [13,29] respectively.

CF is a very important parameter since it defines the prickle factor in fabrics and garments [2,52]. The mean CF value (79.03%) was lesser than 83.9% obtained in Catamarca and similar to 80.8% reported in South and Central Argentina by Mueller et al. [6]. Wurzinger et al. [51] and Laime Huarcaya et al. [45], reported CF values of 89% in Bolivian and Peruvian llamas. Cervantes et al. [34] obtained 84.32% in Peruvian alpacas.

It is necessary to underline that selection of individuals for fibre sampling was oriented in order to cover the full range of variability of llama MFD present in the Puna region of Argentina. It is understandable that reference data in this study differ from those found in the literature. Although the objective of this work is not a comparison between reference values, data from other authors were introduced in order to give a comparative framework to the results obtained.

### 4.2. Spectral Pretreatments and Calibration Models

According to the ANOVA analysis, sample carding improves the prediction accuracy of calibration models. This suggests that untangling and homogenising the fibre improves spectral repeatability. This can be correlated to previous works on the influence of carding treatment on spectral repeatability reported by Amorena et al. [53]. Moreover, Cozzolino et al. [54] obtained more accurate models when using clean wool rather than greasy wool samples for MFD prediction. It is suggested that scattering caused by light reflecting from a sample surface is one of the main restrictive factors for calibration accuracy [21]. This, seen as unfortunate and undesirable in most cases, becomes a useful feature in giving an estimate of the MDF, since this behaviour can be related to thickness and fibre morphology [55,56].

In this work, the best calibration models were obtained without multiplicative (NONE) and none or first derivative (0-0-1-1 or 1-5-3-1) pretreatments. Several publications [13,28,29,56] in the MFD prediction of wool and alpaca fibre reported that the best models were obtained without multiplicative treatments. The model with the highest coefficient of determination (R^2^ = 0.68) was better than the calibrations performed on greasy sheep wool (R^2^ = 0.42) [54]. Nevertheless, results were lower than those obtained in alpaca fibre by Canaza-Cayo et al. (R^2^ = 0.86) [13] and Gishen and Cozzolino (R^2^ = 0.88) [29]. Alomar et al. [28] and Cozzolino et al. [54] obtained better results for raw wool (R^2^ = 0.94) and clean sheep wool, (R^2^ = 0.90), respectively. SECV = 1.96 µm was lower than reported in alpaca by Gishen and Cozzolino (SECV = 2.62 µm) [29] and greasy sheep wool (SECV = 11.2 µm) [54]. However, Alomar et al. and Canaza-Cayo et al. obtained lower values (SECV = 1.24 µm and 1.01 µm, respectively) [13,28]. The value of RPD = 1.91, close to 2, means that models obtained could be implemented for ranking purposes in selection and breeding programmes in llama herds [29,44].

The main limitation found in this study is related to the heterogeneity of fibres. Although carding treatment can improve heterogeneity, further research is proposed to explore replacing the current sample holder with a smaller one in order to reduce light scattering by decreasing sample size. A lack of samples with extreme MFD values (as mentioned in Section 3.1) is also considered a limitation. However, it is very complex to find samples whose values lie within these intervals. Finally, considering research found on similarly heterogeneous materials, such as soils [57,58] or meat [59], it is worth exploring different regression methods involving non-linear approaches, such as artificial neural networks, support vector machines and/or local algorithms.

Research on these issues will be developed in forthcoming papers.

## 5. Conclusions

In this study, the feasibility of NIR spectroscopy to predict quality parameters in llama fibres was evaluated. Models with the highest predictive performance were obtained with no or minimal spectral pretreatment. According to these results the models could be used for screening programs as a sustainable, fast and low-cost method to improve fibre quality. However, this technique still remains elusive and requires further investigation to improve accuracy.

Data presented in this work are encouraging as they contribute to the valorisation of llama fibre and the sustainable development of the textile industry in the Puna territory of Argentina.

In addition, this work is a contribution towards addressing the scarcity of literature related to the use of new technologies for the quality analysis and added value of llama fibre.

## Figures and Tables

**Table 1:** Reference analysis of llama samples obtained with OFDA 2000.

Reference Variables	Mean± SD	Minimum	Maximum	CV
MFD (µm)	25.16 ± 3.75	17.82	37.71	14.93
SDMFD (µm)	8.43 ± 1.83	5.12	13.62	21.76
CVMFD (%)	33.46 ± 4.03	22.7	45.7	12.05
CF (%)	79.03 ± 13.21	24.4	97.3	16.71

MFD: mean fibre diameter; SDMFD: standard deviation of MFD; CVMFD: coefficient of variation of MFD; CF: comfort factor. SD: standard deviation of mean values.

**Table 2:** Statistical variables of MDF prediction models in llama fibre samples.

Model ID	Sample Treatment ^1^	Spectral Range	Pretreatments		Loadings	R^2 2^	SECV ^3^	SEV ^4^	RPD^5^
			Multiplicative	Derivative					
01	Control ^A^	Vis–NIR	NONE	0-0-1-1	5	0.53	2.372	2.672	1.58
10		NIR	NONE	0-0-1-1	5	0.59	2.236	2.547	1.68
19		Discrete	NONE	0-0-1-1	5	0.57	2.286	2.504	1.64
29	Carded ^B^	Vis–NIR	NONE	1-5-3-1	5	0.67	1.965	2.235	1.91
37		NIR	NONE	0-0-1-1	8	0.68	2.088	2.067	1.80
46		Discrete	NONE	0-0-1-1	7	0.64	2.210	2.007	1.70

^1^ ANOVA: different letters (^A,B^) show significant differences (*p* < 0.05). ^2^ R^2^: coefficient of determination in cross validation. ^3^ SECV: standard error of cross validation (µm). ^4^ SEV: standard error of validation (µm). ^5^ RPD: residual predictive value (sd/SECV).

## Data Availability

The data presented in this study are available in Supplementary Material. Additional data other than that are available on request from the corresponding author due to the unavailability of a public repository for this type of data by the institution to which this author belongs.

## Supplementary Materials

The following are available online at https://www.mdpi.com/article/10.3390/ani11071998/s1, Table S1: Statistical variables of MDF prediction models in llama fibre samples.
